# A rare mechanism of ectopy

**DOI:** 10.1007/s12471-020-01425-x

**Published:** 2020-05-11

**Authors:** D. Wesselius, J. Constandse, A. D. Hauer

**Affiliations:** 1 Haga Teaching Hospital, the Hague, The Netherlands; 2 Reinier de Graaf Ziekenhuis, Delft, The Netherlands

A 24-year-old man without relevant past medical history presented with palpitations. He had no other complaints. He took no medication and his family history was negative for cardiac diseases. Physical examination did not reveal any abnormalities except irregular heart beats at auscultation. Electrocardiography (Fig. 1) and Holter monitoring showed frequent occurrence of broad QRS complexes (24%). Exercise testing showed a reduction of these broad QRS complexes during exercise and no signs of ischaemia. Echocardiography and cardiac magnetic resonance imaging (MRI) showed a structurally normal heart with normal left ventricular ejection fraction without signs of fibrosis. What is the underlying mechanism of this arrhythmia?

**Fig. 1:**
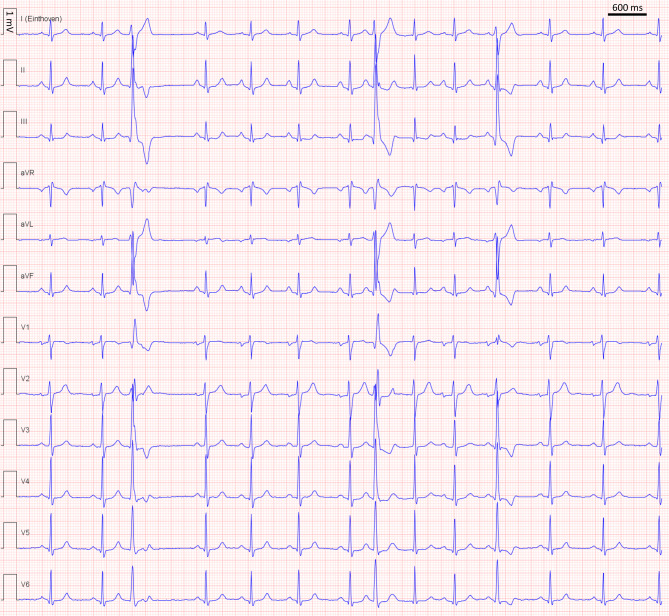
12-lead electrocardiogram during palpitations

## Answer

You will find the answer elsewhere in this issue.

